# Increased cellular senescence in doxorubicin-induced murine ovarian injury: effect of senolytics

**DOI:** 10.1007/s11357-023-00728-2

**Published:** 2023-01-17

**Authors:** Yueyue Gao, Tong Wu, Xianan Tang, Jingyi Wen, Yan Zhang, Jinjin Zhang, Shixuan Wang

**Affiliations:** 1 Department of Obstetrics and Gynecology, Tongji Hospital, Tongji Medical College, Huazhong University of Science and Technology, Wuhan, 430030 Hubei China; 2 National Clinical Research Center for Obstetrical and Gynecological Diseases, Wuhan, 430030 Hubei China; 3 Key Laboratory of Cancer Invasion and Metastasis, Ministry of Education, Wuhan, 430030 Hubei China

**Keywords:** Ovarian injury, Doxorubicin, Cellular senescence, Senolytics, Apoptosis, Fibrosis

## Abstract

Ovarian injury caused by chemotherapy can lead to early menopause, infertility, and even premature senility in female cancer patients, impairing the quality of life and overall health of the cancer survivors seriously. However, there is still a lack of effective protection strategies against such injury. Cellular senescence can be induced by chemotherapeutic agents in multiple organs and may corrode the structure and function of normal tissues. We hypothesized that the widely used first-line chemotherapy drug, doxorubicin, could increase senescent cell burden in normal ovarian tissue during the therapeutic process and that elimination of senescent cells with senolytics would ameliorate doxorubicin-induced ovarian injury. Here, we demonstrated an accumulation of cellular senescence in doxorubicin-treated ovaries through detecting p16 and p21 expression levels and senescence-associated β-galactosidase (SA-β-gal) activity as well as senescence-associated secretory phenotype (SASP) factors. Short-term intervention with the classic senolytic combination dasatinib and quercetin (DQ) or fisetin significantly reduced the load of senescent cells in ovaries after doxorubicin treatment. However, neither DQ nor fisetin alleviated doxorubicin-related ovarian dysfunction. Further experiments showed that ovarian apoptosis and fibrosis following doxorubicin exposure could not be improved by senolytics. Collectively, our study shows that senolytic treatment can eliminate accumulated senescent cells, but cannot reverse the massive follicle loss and ovarian stromal fibrosis caused by doxorubicin, suggesting that cellular senescence may not be one of the key mechanisms in doxorubicin-induced ovarian injury.

## Introduction

The rapid development of modern medicine has made it possible for many cancers to be detected and treated at an early stage, prolonging the survival time of cancer patients significantly [1]. For example, the overall 5-year survival rates for acute lymphoblastic leukemia and breast cancer, the most common malignancies in female children and women of reproductive age respectively, have both exceeded 70% up to now [2–4]. Furthermore, the age of onset of multiple cancers is decreasing. Consequently the quality of post-treatment life and reproductive function of female cancer survivors are receiving increasing attention.

At present, chemotherapy is still the backbone of cancer treatment and doxorubicin is one of the most widely prescribed first-line chemotherapy drugs. As recommended by the National Comprehensive Cancer Network guidelines, doxorubicin is frequently used in various multi-agent chemotherapy regimens for acute lymphoblastic leukemia, Hodgkin lymphoma and invasive breast cancer [5–7]. The systemic side effects of doxorubicin have been extensively studied, and there is considerable evidence that female gonads are vulnerable to doxorubicin [8]. The main clinical manifestations of doxorubicin-related ovarian injury include impaired ovarian reserve, amenorrhea, early menopause, subfertility, and even infertility [9, 10]. The damage mechanisms revealed by previous studies include irreversible DNA damage, increased apoptosis, primordial follicle overactivation, and enhanced oxidative stress [11–13]. Protectants targeting these mechanisms have been developed using mouse models, but these agents had limited effects and have not been tested for their ability to protect against ovarian injury in humans [13, 14]. General fertility preservation strategies, such as embryo and oocyte cryopreservation, provide confined benefits and have many limitations [15, 16]. Therefore, new drugs and mechanisms of damage need to be explored.

Cellular senescence is a cell state in response to different stresses, characterized by irreversible cell cycle arrest, persistent DNA damage, apoptosis resistance, metabolic reprogramming, and senescence-associated secretory phenotype (SASP) [17]. Cellular senescence occurs in normal aging process, and can also be elicited by a variety of stress stimuli, including acute and chronic tissue injury, radiation, and chemotherapy. Excessive and abnormal accumulation of senescent cells in tissues can impair tissue function and regenerative potential, and create a pro-inflammatory milieu which contributes to senescence, dysfunction, or malignant transformation of surrounding normal cells [18]. Senolytics have been developed to selectively kill senescent cells and diminish detrimental impacts of senescence [19]. The combination of dasatinib and quercetin (DQ) is the most typical senolytics. A large number of studies have confirmed its efficacy in clearing senescent cells and its potential to extend healthspan and ameliorate age-related diseases [20–22]. In addition, fisetin, an antioxidant agent with multiple pharmacological functions, was also identified to have senolytic effects in vivo and in vitro [23, 24]. More and more studies have demonstrated that chemotherapy drugs such as doxorubicin and cisplatin can induce cellular senescence in normal tissues like the heart and kidney while playing anti-tumor roles, which may be involved in the pathogenesis of tissue dysfunction [25–27]. To date, it remains unclear whether senescent cells increase in the ovary after doxorubicin treatment and whether senolytics can alleviate doxorubicin-induced ovarian damage.

In this study, we developed an ovarian injury model by doxorubicin in mice, and confirmed that doxorubicin treatment increased cellular senescence in murine ovaries. However, neither DQ nor fisetin could improve the ovarian reserve and fertility of doxorubicin-treated mice, although both significantly reduced ovarian senescence. Further experiments showed that DNA damage, apoptosis, and stromal fibrosis caused by doxorubicin could not be reversed through DQ or fisetin intervention. Our results suggest that cellular senescence may not be a key mechanism of doxorubicin-induced ovarian injury and, therefore, senolytics may not be an ideal choice for fertility protection in female cancer patients treated with doxorubicin-containing regimens.

## Materials and methods

### Animal care and drug administration

Female C57BL/6J mice of 7-week-old were purchased from Beijing Vital River Laboratory Animal Technology Co., Ltd. and fed freely for 1 week for environmental adaptation before drug administration. All the mice were housed in ventilated cages with free access to food and water. The feeding environment was maintained at a temperature of 22 ± 2 °C and a humidity of 55% ± 15% on a 12:12 h light/dark cycle.

The first batch of animal experiments included 2 groups (with 16 female 8-week-old C57BL/6J mice in each group): control group (Con) and doxorubicin group (Dox). Doxorubicin (D8740, Solarbio, China) was intraperitoneally injected once at a dose of 10mg/kg. The second batch of mice were divided into six groups (with 20 female 8-week-old C57BL/6J mice in each group): vehicle control group (Vehicle), doxorubicin group (Dox), doxorubicin plus DQ group (Dox + DQ), doxorubicin plus fisetin group (Dox + F), DQ group, and Fisetin group. DQ (dasatinib, 5 mg/kg; quercetin, 50 mg/kg) or fisetin (100 mg/kg) or vehicle solution (10% polyethylene glycol 400, PEG400) was administered by oral gavage once every other day for 3 weeks [24, 28]. A single intraperitoneal injection of doxorubicin (10 mg/kg) or saline was given at the day after the second administration of senolytics. Senolytic drugs and solvent used in this study are commercially available from MedChemExpress (dasatinib, HY-10181; quercetin, HY-18085; fisetin, HY-N0182; PEG400, HY-Y0873A).

Blood samples and ovaries of female mice were collected at 11 weeks of age. Body weight and ovary weight were recorded. Six ovaries from each group were preserved in 4% paraformaldehyde for subsequent process. Three ovaries from each group were flash-frozen in liquid nitrogen to make frozen sections and the other ovaries were stored at −80 °C for protein or RNA extraction.

### Estrous cycle monitoring

Vaginal smears of mice were taken at about 9–11 a.m. every day for consecutive 2 weeks. Vaginal cells procured by washing the vagina with saline were spread on clean glass slides and stained with hematoxylin and eosin (H&E). The stage of the estrous cycle was judged by the cytological characteristics under a microscope as previously described [29]. Mice were categorized as regularly cycling if they consistently displayed cycles that were 4–6 days long. Mice with mean cycle lengths out of this range and mice with cycles of variable lengths or without apparent cycles were categorized as irregularly cycling [30].

### Serum hormone measurement

Blood samples of female mice were collected at diestrus. Levels of serum anti-müllerian hormone (AMH), follicle-stimulating hormone (FSH), and estradiol (E2) were measured by enzyme-linked immunosorbent assay according to the manufacturer’s instructions (CSB-E13156m, CSB-06871m, and CSB-E05109m; Cusabio Technology LLC, USA).

### Follicle counting

The ovaries fixed in 4% paraformaldehyde were embedded in paraffin and then cut into pieces of 5 μm, with four consecutive pieces mounted on one glass slide. Every fourth slide was stained with H&E and analyzed under a light microscope by 2 persons who were blinded to the group of the sections. Follicles without visible oocytes were excluded while zona pellucida remnants (ZPRs) were classified as late atretic follicles. The detailed procedures were performed as previously described [31].

### Mating test

In the second batch of animal experiments, six female mice from each group were randomly selected for mating test. Two female mice and one sexually mature male mouse were kept in a cage for free mating. After 10 days, the female mice were placed in solitary cages. Then, the pregnancy status, parturition outcome, litter size, and pup conditions were monitored and recorded. The mating test was repeated twice. Two rounds of mating test were 50 days apart to make sure all mice were weaned before the second round.

### Senescence-associated β-galactosidase (SA-β-gal) staining

SA-β-gal staining of frozen ovarian tissue sections were performed using a commercial SA-β-gal staining kit (C0602, Beyotime Biotechnology, China) according to the manufacture’s protocol. In brief, frozen sections of ovarian tissue were rewarmed at room temperature, soaked with PBS, and then fixed in the fixative solution for 15 min. After washing with PBS for three times, samples were incubated with SA-β-gal working solution at 37 °C for 16 h. Ice-cold PBS was used to stop the enzymatic reaction.

### Immunohistochemistry

Three representative ovarian sections in each group were selected for immunohistochemical analysis. After deparaffinization and rehydration, the sections were heated in Tris-Ethylene Diamine Tetraacetic Acid buffer (pH 9.0) for 15 min at 95 °C for antigen retrieval. Next, naturally cooled sections were washed three times with PBS, and incubated in 3% H2O2 solution for 30 min at room temperature. The ovarian sections were blocked in 3% BSA for 1 h and then incubated with primary antibody (p16 #A0262, ABclonal Technology, China; p21 #A11454, ABclonal Technology, China; γH2AX #AP0687, ABclonal Technology, China) overnight at 4 °C. The next day the sections were incubated with secondary antibody (HRP-conjugated Goat Anti-Rabbit IgG (H+L), GB23204, Servicebio, China) for 50 min at room temperature followed by 3 washes with PBS. Subsequently, the sections were visualized with DAB chromogenic agent (AR1022, Boster, China) and counterstained by hematoxylin. Images were acquired using the cellSens Dimension software (Olympus Soft Imaging Solutions GmbH, Germany) under a light microscope and the relative expression was evaluated by Image Pro Plus software [32]. Briefly, we used the straw tool in HSI mode to determine the region for analysis and obtained the integrated optical density (IOD) and area of each image. The mean density was calculated by IOD/Area to represent the corresponding antigen expression level. All images were analyzed under the same analytical environment and color parameters.

### Immunofluorescence

The expression levels of typical fibrosis biomarker, alpha-smooth muscle actin (α-SMA), in ovaries were detected by immunofluorescence staining according to the routine procedure [33]. Ovarian sections were incubated with primary antibody (α-SMA #ab124964, Abcam, Cambridge, UK) overnight at 4 °C. The next day, after incubation with secondary antibody (Cy3 conjugated Goat Anti-Rabbit IgG (H+L), GB21303, Servicebio, China) for 60 min at 37 °C, nuclei were counterstained with 4′, 6-diamidino-2-phenylindole (DAPI) (G1012, Servicebio, China). Images were taken by a fluorescence microscope (Olympus).

### Terminal deoxynucleotidyl transferase-mediated dUTP nick-end labeling (TUNEL) assay

The cell apoptosis in murine ovaries was analyzed using the One Step TUNEL Apoptosis Assay Kit (C1088, Beyotime Biotechnology, China). According to the manufacturer’s instruction, ovarian sections were first permeabilized by Proteinase K at room temperature for 15 min and then incubated with TUNEL reaction mixture at 37 °C for 1 h. The nuclei were counterstained with DAPI. For each sample, 3–5 images were taken from random fields.

### Sirius red staining

A series of representative ovarian tissue sections were routinely dewaxed to water and stained with Sirius red dye for 15 min. After dehydration, ovarian sections were sealed with neutral resin and photographed for quantitative analysis.

### Western blot

Total protein was extracted from ovaries as the routine procedure and separated by 10% or 12% sodium dodecyl sulfate polyacrylamide gel electrophoresis. Proteins in the gel were subsequently transferred to polyvinylidene fluoride (PVDF) membranes. After blocking with 5% skimmed milk for 1 h, membranes were incubated with specific primary antibody (p16 #A11337, ABclonal Technology, China; p21 #PA9426, Abmart, China; and β-actin #AC026, ABclonal Technology, China) overnight at 4 °C. The next day PVDF membranes were washed 3 times with Tris Buffered Saline plus Tween-20 and incubated with appropriate secondary antibody for 1 h at 37 °C. After another 3 washes, target blots were imaged using the chemiluminescence method. Quantification of the protein expression was conducted with Image Lab software based on the integrated light intensity of each band. β-actin expression was measured to verify equal loading.

### Quantitative real-time polymerase chain reactions (qRT-PCR)

Total RNA from ovaries was extracted and purified using RNAiso plus reagent (Takara, Nojihigashi, Japan). Samples containing 2 g of total RNA were DNase-treated with gDNA wiper (R223-01, Vazyme, China) and then reverse transcribed into cDNA using HiScript reverse transcriptase (R223-01, Vazyme, China). Real-time PCR was performed on a CFX96 real-time PCR system (Bio-Rad, USA) at a final volume of 40 μl. The *Actb* gene was used as endogenous control to calculate relative gene expression based on the comparative CT method. The primer sequences are listed in Table 1.

**Table 1:** The sequences of primers used for qRT-PCR assay

Gene	Forward primer (5′-3′)	Reverse primer (5′-3′)
*Cdkn2a*	GAACTCTTTCGGTCGTACCC	CGAATCTGCACCGTAGTTGA
*Cdkn1a*	TTGTCGCTGTCTTGCACTCT	TCTCTTGCAGAAGACCAATC
*Il6*	TGAACAACGATGATGCACTTG	CTGAAGGACTCTGGCTTTGTC
*Mcp1*	CTACCTTTTCCACAACCACCTC	ATTAAGGCATCACAGTCCGAGT
*Tgfβ1*	CACCATCCATGACATGAACC	TGGTTGTAGAGGGCAAGGAC
*Bax*	CAGGATGCGTCCACCAAGAA	GCAAAGTAGAAGAGGGCAACCA
*Bcl2*	TGGAGAGCGTCAACAGGGAGA	GCCAGGAGAAATCAAACAGAGGT
*Caspase 3*	AGCAGCTTTGTGTGTGTGATTCTAA	AGTTTCGGCTTTCCAGTCAGAC
*Acta2*	CTGACAGAGGCACCACTGAA	CATCTCCAGAGTCCAGCACA
*Col1a1*	TTCTCCTGGCAAAGACGGACTCAA	GGAAGCTGAATCATAACCGCCA
*Timp1*	TCATGGAAAGCCTCTGTGGAT	CGGCCCGTGATGAGAAACT
*Timp2*	GCGTTTTGCAATGCAGACG	ATTCCCGGAATCCACCTCC
*Mmp2*	TTTGCTCGGGCCTTAAAAGTAT	CCATCAAACGGGTATCCATCTC
*Actb*	ATGACCCAAGCCGAGAAGG	CGGCCAAGTCTTAGAGTTGTTG

### Statistical analysis

Data are presented as mean ± SD and a *p* < 0.05 was considered significantly different. Statistical comparisons were performed using unpaired Student’s *t*-tests, Chi-square test, or one-way ANOVA. Statistical analyses were performed in SPSS 26.0 and GraphPad Prism 8.0.

## Results

### Accumulation of senescent cells in murine ovaries after doxorubicin treatment

**Fig. 1:**
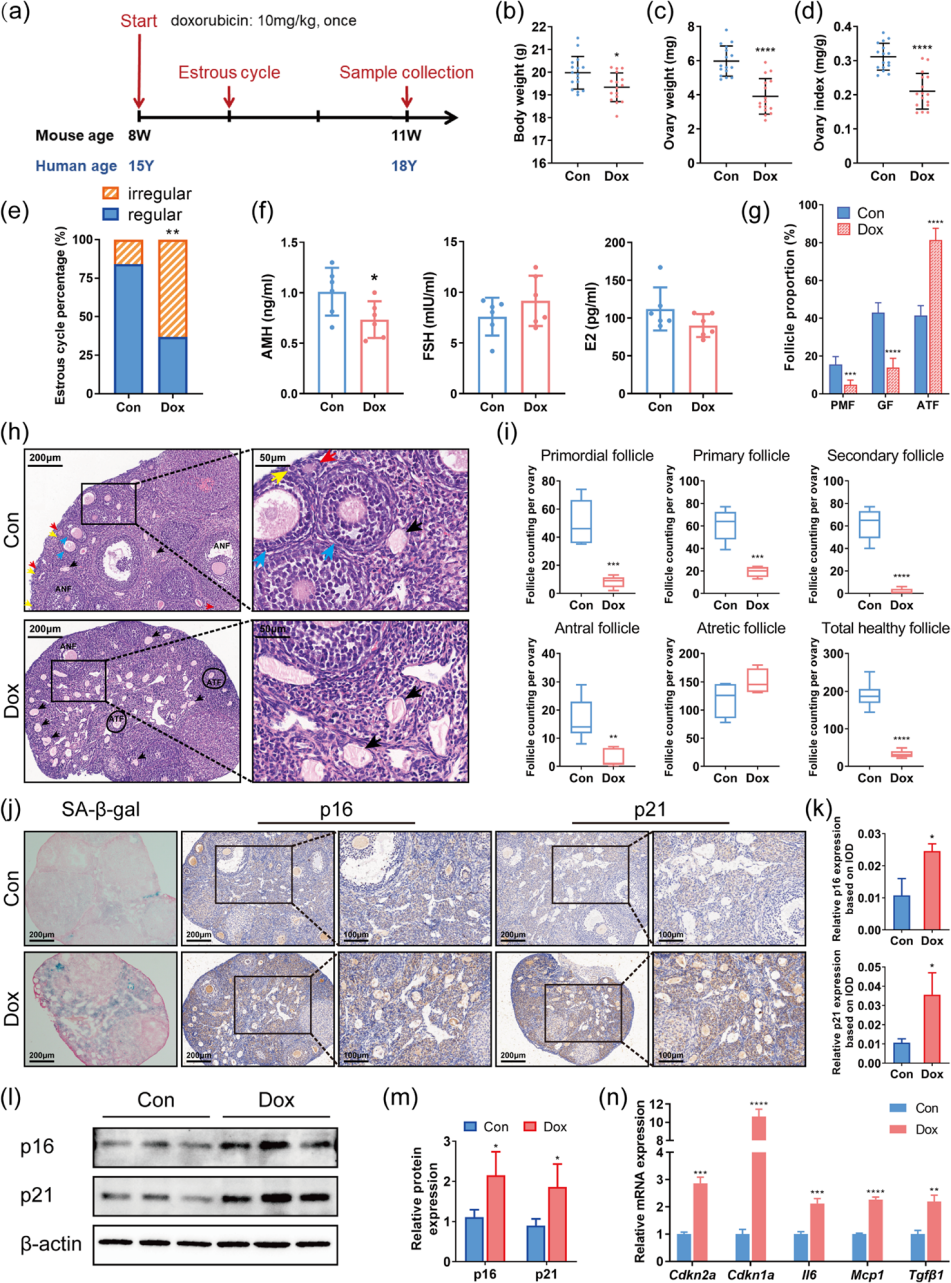
Ovarian senescence is increased in doxorubicin-treated mice. **a** Schematic diagram for animal experiment design. **b**–**d** Body weight, ovary weight, and ovary index of mice after saline or doxorubicin injection (*n* = 16, unpaired Student’s *t*-test). **e** The proportion of regular or irregular estrous cycles after saline or doxorubicin injection (*n* = 16, Chi-square test). **f** The serum levels of AMH, FSH, and E2 at diestrus (*n* = 6, unpaired Student’s *t*-test). **g** The proportion of follicles at different stages (PMF: primordial follicles, GF: growing follicles, ATF: atretic follicles). **h** Representative H&E staining images of murine ovaries. Yellow arrows: primordial follicles, red arrows: primary follicles, blue arrows: secondary follicles, black arrows: zona pellucida remnants. ANF: antral follicles. **i** Follicle counting results based on H&E staining of serial ovarian sections (*n* ≥ 5, unpaired Student’s *t*-test). **j** Representative images of SA-β-gal staining and IHC detection of p16 and p21 in the murine ovaries. **k** IHC scores of relative expression of p16 and p21 based on IOD (*n* = 3, unpaired Student’s *t*-test). IOD: integrated optical density. **l**, **m** Protein expression of p16 and p21 in ovarian tissues detected by western blot. **n** Relative mRNA expression of genes related to cellular senescence and SASP (*n* = 3, unpaired Student’s *t*-test). Data are presented as mean ± SD. *p < 0.05, **p < 0.01, ***p < 0.001, ****p < 0.0001

We treated 8-week-old female mice with clinically relevant dose of doxorubicin at 10mg/kg once (Fig. 1a) [25]. Compared to the saline control group, doxorubicin-treated mice had lower body weight, ovary weight, and ovary index, i.e., the ratio of ovary weight to body weight (Fig. 1b–d). Estrous cycle monitoring and serum hormone examination were performed to reflect ovarian endocrine function. Dox group (see Materials and methods for simplified group name) had a higher percentage of irregular estrous cycles (Dox group 63.16% vs. Con group 15.79%, *p*<0.01, Fig. 1e). Serum AMH levels decreased significantly after doxorubicin administration, while E2 and FSH showed a tendency to decrease and increase, respectively (Fig. 1f). In addition, H&E staining and subsequent ovarian follicle counting were conducted to present pathological alternations of ovarian reserve more directly (Fig. 1h). In doxorubicin-treated mice, primordial follicles (PMF), primary follicles, secondary follicles, and antral follicles were largely reduced, while atretic follicles (ATF) had an increasing tendency without statistical significance (Fig. 1i). Primary, secondary, and antral follicles were further classified as growing follicles (GF). The proportion of PMF and GF decreased dramatically while the proportion of ATF increased remarkably in doxorubicin-treated ovaries (Fig. 1g). Therefore, doxorubicin of clinically relevant dose can cause ovarian endocrine dysfunction and ovarian reserve depletion in female mice.

Next, to determine whether cellular senescence was induced in the ovaries after doxorubicin injection, we conducted SA-β-gal staining and evaluated the expression of biomarkers of cellular senescence. SA-β-gal positivity, which is upregulated in senescent cells [34], was greater in ovaries of Dox group compared to control ovaries, especially in ovarian stroma (Fig. 1j). Immunohistochemistry (IHC) staining of the cyclin-dependent kinase inhibitors (CDKIs) p16 and p21, another two typical markers of cellular senescence [34], showed consistent changes with SA-β-gal staining (Fig. 1j, k). Protein expression of p16 and p21 in ovarian tissues further detected by western blot was also enhanced following doxorubicin treatment (Fig. 1l, m). Moreover, mRNA levels of *Cdkn2a* and *Cdkn1a*, genes coding p16 and p21 respectively, along with several SASP genes including *Il6*, *Mcp1*, and *Tgfβ1*, were elevated significantly in doxorubicin-treated ovaries (Fig. 1n). Thus, these results suggested that doxorubicin treatment can indeed induce accumulation of senescent cells in the ovaries.

### Non-alleviation of doxorubicin-induced ovarian injury by senolytics

**Fig. 2:**
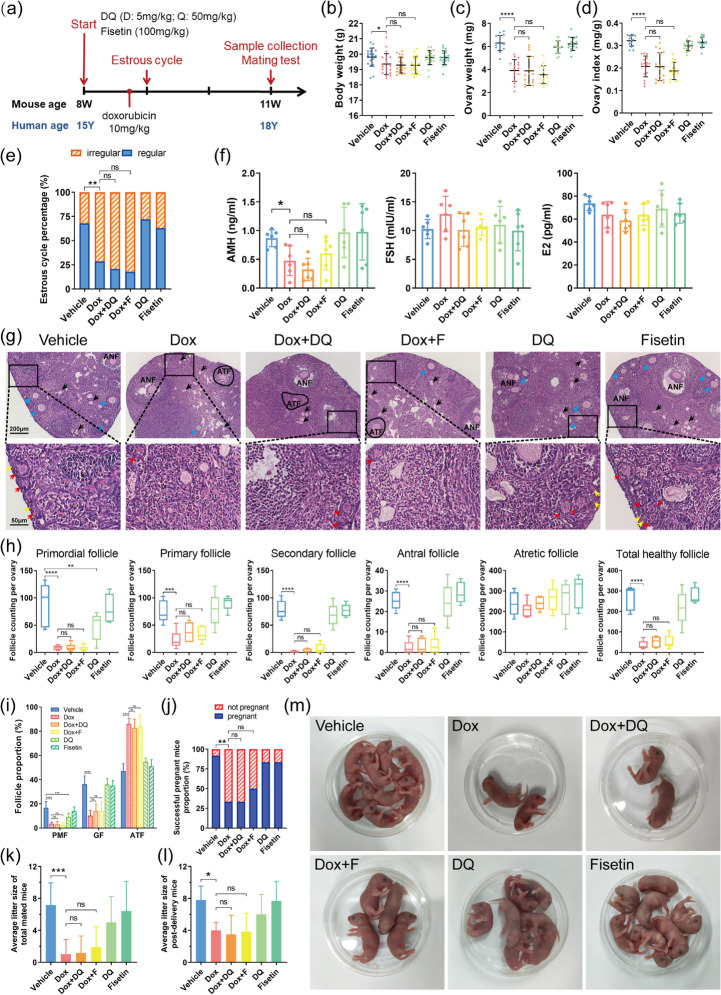
Senolytics cannot alleviate doxorubicin-induced ovarian injury. **a** Brief schematic diagram for animal experiment design. **b**–**d** Body weight, ovary weight, and ovary index of mice in each group (*n* ≥ 16, one-way ANOVA). **e** The proportion of regular or irregular estrous cycles in each group (*n* ≥ 18, Chi-square test). **f** The serum levels of AMH, FSH, and E2 at diestrus (*n* = 6, one-way ANOVA). **g** Representative H&E staining images of murine ovaries from each group. Yellow arrows: primordial follicles, red arrows: primary follicles, blue arrows: secondary follicles, black arrows: zona pellucida remnants. ANF: antral follicles, ATF: atretic follicles. **h** Follicle counting results according to H&E staining of serial ovarian sections (*n* ≥ 5, one-way ANOVA). **i** The proportion of follicles at different stages (PMF: primordial follicles, GF: growing follicles). **j** The proportion of successful pregnant mice in the mating test (*n* = 12, Chi-square test). **k** Average litter size of total mated mice (*n* = 12, one-way ANOVA). **l** Average litter size of post-delivery mice (*n* = 3~11, one-way ANOVA). **m** General state of pups in different groups. Data are presented as mean ± SD. *p < 0.05, **p < 0.01, ***p < 0.001, ****p < 0.0001

Given the considerable increase in cellular senescence in the ovaries of doxorubicin-treated mice, we hypothesized that intervention with senolytics capable of obliterating senescent cells to female mice would ameliorate ovarian injury induced by doxorubicin. In present study, the most classic senolytic cocktail DQ and the potential senolytic drug fisetin were selected and administered intragastrically before and after doxorubicin injection (Fig. 2a). Intriguingly, in groups subjected to doxorubicin, treatment with DQ or fisetin did not mitigate doxorubicin-induced changes in body weight, ovary weight/index, estrous cycles, serum hormone levels, and ovarian follicle reserve (Fig. 2b–i). Besides, mating tests revealed that doxorubicin greatly damaged the fertility of female mice, while DQ or fisetin intervention did not improve the successful pregnancy rate and average litter size of doxorubicin-treated mice (Fig. 2j–m). It is of note that DQ alone or fisetin alone had no evident effect on most of the aforementioned tests compared to the vehicle control group, except that DQ alone significantly reduced the number and proportion of PMF in murine ovaries (Fig. 2h, i).

### Elimination of senescent cells by senolytics in doxorubicin-treated ovaries

**Fig. 3:**
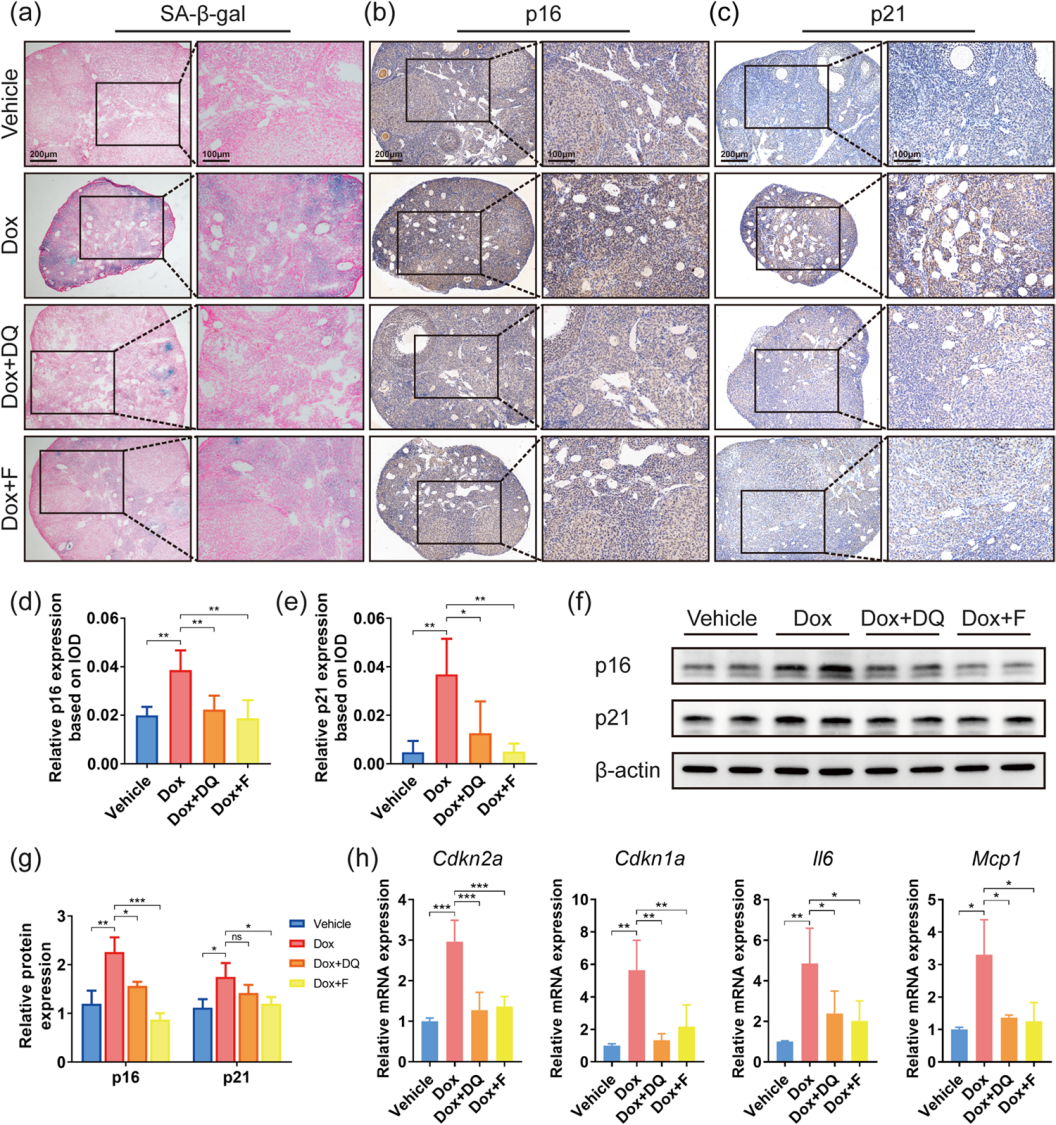
Senolytics can reduce ovarian senescence in doxorubicin-treated mice. **a** Representative SA-β-gal staining images of murine ovarian sections. **b**, **c** Representative images of p16 and p21 expression in murine ovaries by IHC. **d**, **e** IHC scores of relative expression of p16 and p21 based on IOD (*n* = 3, one-way ANOVA). **f**, **g** Protein expression of p16 and p21 in ovarian tissues shown by western blot. **h** Relative mRNA expression levels of genes related to cellular senescence and SASP in ovaries (*n* = 3, one-way ANOVA). Data are presented as mean ± SD. *p < 0.05, **p < 0.01, ***p < 0.001, ****p < 0.0001

To figure out whether the two senolytics eliminated senescent cells accumulated in ovaries injured by doxorubicin, we detected cellular senescence biomarkers in each group. Compared with the Dox group, both DQ and fisetin intervention shrank the SA-β-gal staining positive area remarkably (Fig. 3a) and reduced the upregulated p16 and p21 expression to the level of control ovaries (Fig. 3b–e). As shown by western blot, DQ and fisetin decreased the protein levels of p16 and p21 in ovarian tissues following doxorubicin injection (Fig. 3f, g). Additionally, treatment with DQ or fisetin downregulated the mRNA levels of *Cdkn2a*, *Cdkn1a*, *Il6*, and *Mcp1*, but not *Tgfβ1* (Figs. 3h and 5e). Together, these results demonstrated that short-term administration of DQ or fisetin was effective in counteracting doxorubicin-induced increase in ovarian senescence.

### No improvement in ovarian apoptosis and fibrosis upon senolytic treatment

**Fig. 4:**
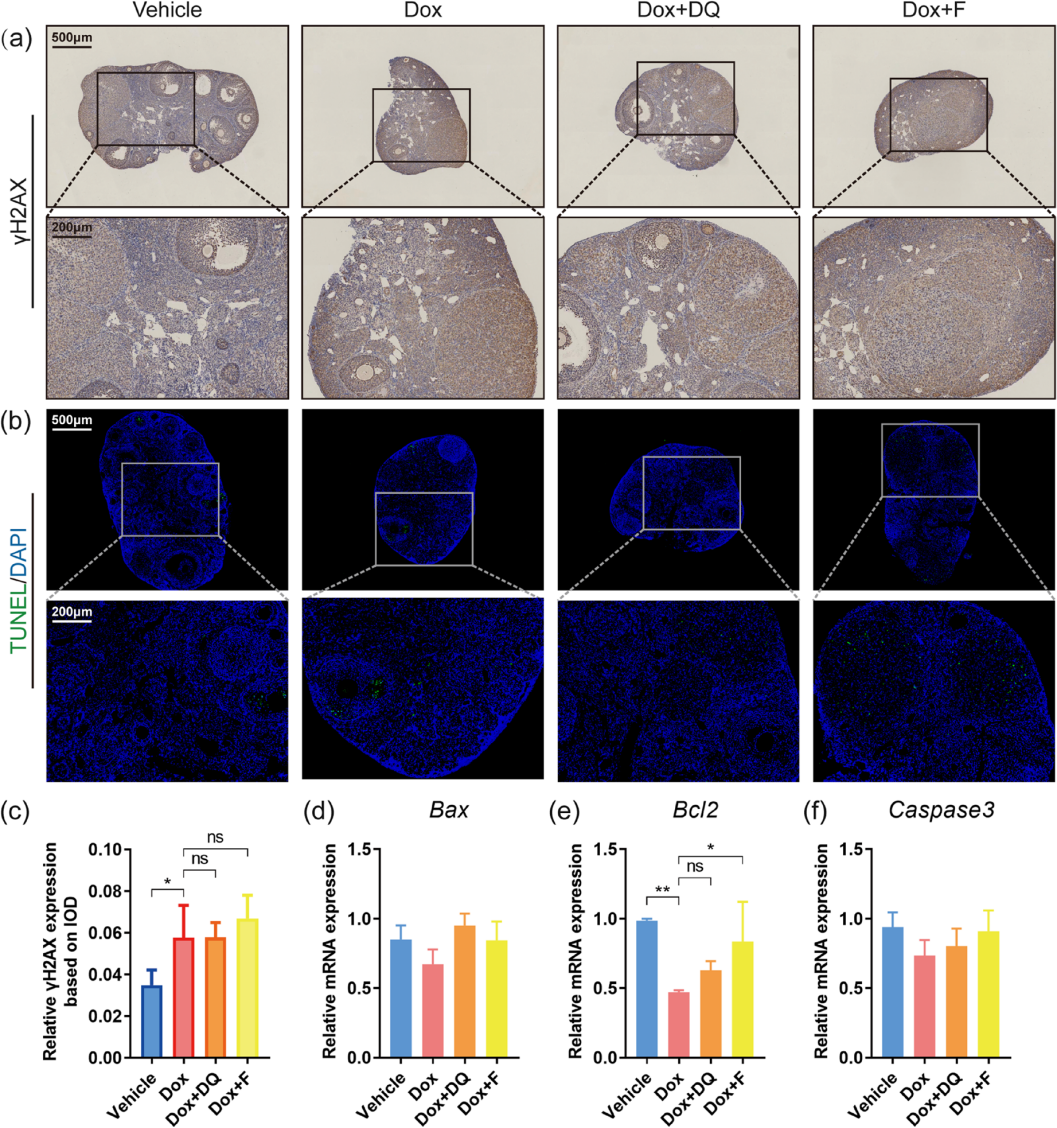
Senolytics have no protective effect against ovarian apoptosis after doxorubicin treatment. **a**, **c** Expression of DNA damage marker γH2AX in murine ovaries detected by IHC (*n* = 3, one-way ANOVA). **b** Representative TUNEL staining images of ovarian sections. Green fluorescent dots indicate TUNEL-positive apoptotic cells. **d**–**f** Relative mRNA expression of apoptosis-related genes in ovaries (*n* = 3, one-way ANOVA). Data are presented as mean ± SD. *p < 0.05, **p < 0.01

To further elucidate why DQ and fisetin failed to rescue ovarian function in doxorubicin-treated mice, we focused on other pathways of damage besides cellular senescence. Apoptosis is the main and the most studied process downstream of DNA damage in doxorubicin-induced ovarian injury [9]. We next conducted immunohistochemistry of γH2AX and TUNEL staining to detect DNA damage and apoptosis in the ovaries. Doxorubicin-treated ovaries showed increased expression of γH2AX but neither DQ nor fisetin reversed this change (Fig. 4a, c). However, TUNEL staining results indicated no obvious change among different groups (Fig. 4b). The mRNA expression of anti-apoptotic gene *Bcl2* in doxorubicin-treated ovaries was significantly lower and elevated by fisetin but not DQ, while the mRNA expression of pro-apoptotic genes *Bax* and *caspase 3* did not differ significantly among the groups (Fig. 4d–f). Given that there was an interval of 2–3 weeks between the injection of doxorubicin and the acquisition of the ovaries, these results are in accordance with previous research which had proved that apoptosis in the ovary peaked at day 1 after doxorubicin treatment and a majority of apoptotic follicles degenerated completely within 2 weeks [12]. Since ovarian apoptosis mainly occurred in granulosa cells, the degeneration and massive loss of apoptotic follicles might concealed our results of apoptosis-related detection. The decrease in the proportion of PMF and GF and the increase in the proportion of ATF in doxorubicin-treated ovaries can be another evidence supporting the occurrence of apoptosis. Apparently, neither DQ nor fisetin exerted an evident protective effect against apoptosis.

**Fig. 5:**
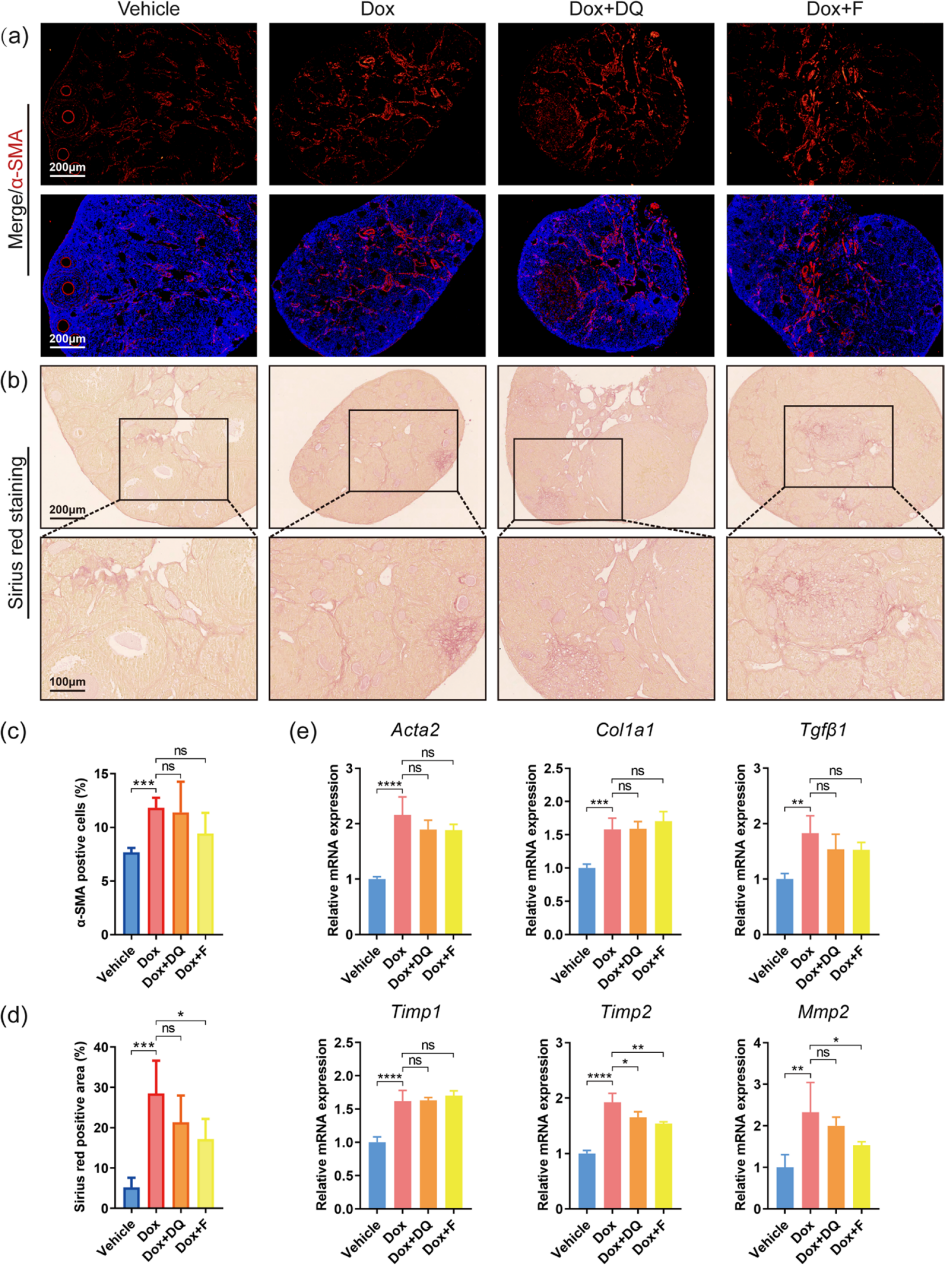
Senolytics cannot prevent ovarian fibrosis after doxorubicin treatment. **a** Immunofluorescence images of ovarian sections stained with α-SMA antibody. Red fluorescence indicates α-SMA-positive cells. **b** Representative images of Sirius red staining in murine ovaries. **c**–**d** The percentage of α-SMA positive cells and Sirius red positive area in ovarian sections (*n* = 3, one-way ANOVA). **e** Relative mRNA expression of fibrosis-related genes in ovaries (*n* = 3, one-way ANOVA). Data are presented as mean ± SD. *p < 0.05, **p < 0.01, ***p < 0.001, ****p < 0.0001

Apart from apoptosis, former studies around doxorubicin have shown that it can induce parenchymal fibrosis and cause tissue dysfunction in different organs [35, 36]. As a marker of activated fibroblasts, α-SMA increases with the severity of tissue fibrosis in multiple organs [33]. We performed α-SMA immunofluorescence staining and Sirius red staining to assess the level of ovarian fibrosis. Doxorubicin obviously aggravated the degree of fibrosis in ovarian stroma (Fig. 5a–d). Both DQ and fisetin could not attenuate the increase in α-SMA (Fig. 5a, c), whereas fisetin slightly but significantly reduced the positive area of Sirius red staining following doxorubicin treatment (Fig. 5b, d). As shown in Fig. 5e, the mRNA expression of *Acta2*, *Col1a1*, *Tgfβ1*, *Timp1*, *Timp2*, and *Mmp2* was significantly higher in doxorubicin-treated ovaries. These genes are involved in tissue remodeling and fibrosis progression. Administration of DQ slightly reversed the upregulation of *Timp2* expression but not the others. Meanwhile, fisetin reduced the mRNA levels of *Timp2* and *Mmp2* in ovaries injured by doxorubicin. Overall, these two senolytics did not prevent ovarian fibrosis effectively after doxorubicin treatment, although some indicators were partly improved.

## Discussion

Chemotherapy is still a common treatment for cancer at present, and the short-term and long-term adverse effects caused by chemotherapy have always been of concern. Chemotherapy-associated ovarian damage not only leads to amenorrhea, early menopause, and infertility in young female cancer patients but also promotes premature aging in cancer survivors and amplify the risk of age-related diseases such as Alzheimer’s disease, cardiovascular disease, and osteoporosis [10, 37, 38]. Existing fertility preservation strategies, such as preventive agents, embryo and oocyte cryopreservation, ovarian tissue cryopreservation, stem cell therapy, and artificial ovary, may benefit women with cancer [15, 16]. But these methods are still not widely used owing to their limited benefits, low success rate, risk of cancer recurrence, or ethical issues. Thus, the exploration of potential drugs to protect the ovaries from chemotherapy damage has profound implications for women’s health. Despite the successive emergence of novel antitumor agents, doxorubicin is still widely used in clinical practice. Previous studies have shown that doxorubicin can deplete ovarian follicle reserve and impair female fertility [8, 9]. The mechanisms of doxorubicin-induced ovarian injury may involve DNA damage, apoptosis, hyperactivation of PMF, and oxidative stress [11–13]. However, few studies have discovered drugs that can effectively prevent doxorubicin-induced ovarian injury.

Recent studies have found that doxorubicin can trigger senescence of tumor cells while playing antineoplastic roles, and can also induce cellular senescence in normal organs and tissues such as the heart, liver, and skin [25, 26, 39, 40]. As a hazard factor that may gradually erode the normal tissue structure and function, cellular senescence has become a potential target for the treatment of chemotherapy-related injuries. However, it is unknown how cellular senescence changes in normal ovarian tissue exposed to doxorubicin. In this study, we demonstrated for the first time that clinically relevant dose of doxorubicin increased senescent cells in murine ovaries. This was evidenced by enhanced SA-β-gal activity, elevated p16 and p21 expression, and increased transcription of several common SASP genes such as *Il6*, *Mcp1*, and *Tgfβ1*. SA-β-gal staining can reflect the lysosomal activity and is arguably the most common senescence biomarker [34]. The expression levels of CDKIs p16 and p21, main drivers of cell cycle arrest, are high in senescent cells [34]. SASP is a set of heterogeneous molecules secreted by senescent cells, which is unspecific but can help to identify cellular senescence. The changes of above biomarkers in our study indicates that doxorubicin can induce cellular senescence in normal ovarian tissue.

We speculated that induced cellular senescence could be a reason for ovarian injury caused by doxorubicin, and eliminating accumulated senescent cells might rescue the ovarian function to an extent. Senolytics are a class of drugs that can selectively kill senescent cells [19]. There is increasing evidence that senolytics have great potential in anti-aging medicine. DQ is the most classic senolytics with a broad-spectrum senescent cell-killing effect. The combination of DQ has been reported to improve physical function and prolong lifespan in old mice, and alleviate a variety of age-related diseases, including cognitive deficits, bone loss, intervertebral disc degeneration, and obesity-mediated metabolic dysfunction [20–22, 41, 42]. In addition, short-term intermittent use of DQ after acute injuries such as chemotherapy-induced lung injury and radiation ulcers mitigated the severity of lesions [43, 44]. Fisetin is another senolytic drug that can extend healthspan of mice [23]. In multiple disease models, such as systemic lupus erythematosus and primary sclerosing cholangitis, fisetin was able to improve the disease phenotypes by reducing the number of senescent cells [45, 46]. Fisetin also protected against tissue damage caused by chemotherapy [24, 47]. Both senolytics are currently in clinical trials and have achieved preliminary results [48, 49]. In our study, these two senolytics were used to intervene doxorubicin-induced ovarian injury in mice. Administration of DQ or fisetin reduced the positive area of SA-β-gal staining and downregulated the expression of p16 and p21 at mRNA and protein levels in doxorubicin-treated ovaries. Increased transcription of SASP genes *Il6* and *Mcp1* in the ovaries was reversed by DQ and fisetin, suggesting that senolytics can relieve pro-inflammatory milieu to some extent. Taken together, both DQ and fisetin significantly reduced ovarian senescence in doxorubicin-treated mice. However, beyond our expectation, removal of senescent cells did not restore the depleted ovarian reserve and declined fertility.

Next we inquired into this discrepancy between senescent cell clearance and recovery of ovarian function by analyzing some other possible mechanisms of injury. DNA damage and apoptosis are the most studied molecular pathways of doxorubicin-induced ovarian damage [9]. Our results showed that DNA damage persisted in ovaries following doxorubicin exposure, and was not attenuated by senolytic treatment. However, there was no obvious difference in ovarian apoptosis among the groups, which may be due to our experimental schedule, that is, murine ovaries were harvested 2-3 weeks after doxorubicin injection, when most apoptotic follicles in the ovaries had completely degenerated [12]. According to H&E staining and follicle counting results, the proportion of PMF and GF in doxorubicin-treated ovaries drastically decreased, whereas the proportion of ATF, especially end-stage atretic follicles (ZPRs), significantly increased. This could be an evidence that apoptosis had occurred in the ovaries and senolytics failed to counteract the outcome of ovarian apoptosis. Besides, tissue fibrosis is another common mechanism in organ dysfunction and structural destruction caused by doxorubicin [35, 36, 50, 51]. Through detecting several representative fibrosis markers, we found that doxorubicin aggravated ovarian stromal fibrosis to a large extent. Neither DQ nor fisetin reversed the progression of fibrosis in ovaries injured by doxorubicin, despite partial improvement in a few indicators. Thus, although senolytics effectively obliterated senescent cells, they were unable to prevent ovarian apoptosis and stromal fibrosis induced by doxorubicin treatment. The former contributes to massive loss of follicle reserve, while the latter can disturb ovarian microenvironment and restrict the growth and development of remaining follicles. This could be the reason why ovarian damage was not rescued by senolytic treatment.

In summary, it is reasonable to speculate that the key mechanisms of doxorubicin-induced ovarian injury may be initial apoptosis and consequent loss of follicle reserve, as well as persistent stromal fibrosis. Cellular senescence may be a concomitant phenomenon in ovarian stroma, rather than a main mechanism of ovarian damage. Therefore, the strategy of eliminating senescent cells with senolytics may not be an optimal choice for protection against doxorubicin-induced ovarian injury. The majority of studies on senolytics have been conducted on very old mice, where chronic accumulation of senescent cells may cause more damage and senolytics may be more beneficial. The subjects of this study were mice aged 2-3 months, corresponding to adolescent females aged 15–18 years. The accumulation of senescent cells in the ovaries after doxorubicin treatment should be an acute response to stress, which had no profound impacts on ovarian reserve. This may also be a reason for the poor protective effects of senolytics. It is worth mentioning that our previous study demonstrated that DQ alone or combined with metformin recovered the ovarian endocrine and reproductive function in cisplatin-exposed mice [52]. Such difference in the efficacy of DQ suggests that the role of cell senescence in ovarian injury varies with antitumor drugs, and the therapeutic potential of senolytics in chemotherapy-associated ovarian damage should not be completely denied. More comprehensive studies are needed to assess the extent of cellular senescence in the ovaries when diverse chemotherapeutic agents are used alone or in combination, and to evaluate the potential benefits of applying senolytics.

Limitations of this study should be emphasized here. First, we did not specify which type of ovarian cells underwent senescence following doxorubicin exposure and whether DQ or fisetin eliminated all types of senescent cells in ovaries. The introduction of single-cell transcriptomics may help answer this question. Second, we performed a short-term intervention with only two senolytics, DQ and fisetin [24, 28]. Although this is sufficient to reduce ovarian senescence induced by doxorubicin, whether longer interventions or other senolytics and senomorphics will be beneficial for doxorubicin-associated ovarian dysfunction is still unclear. Future studies should examine the senolytic and ovary-protective effects of different drugs under varied duration of administration. Third, our study observed that DQ alone reduced the number and proportion of PMF in murine ovaries, but had no obvious influence on other grades of follicles. However, no further experiments were carried out to investigate the underlying mechanisms and possible consequences of this alteration. Considering that dasatinib is also an effective antitumor agent, it is necessary to design experiments to determine if DQ treatment itself is harmful to female gonads. Last, we did not explore additional patterns of damage beyond cellular senescence, apoptosis, and fibrosis; thus, it is not clear whether DQ and fisetin have other effects on the ovaries than obliterating senescent cells.

In conclusion, doxorubicin treatment can induce cellular senescence in murine ovaries and short-term administration of senolytics DQ or fisetin can reduce excessive senescent cells. Nevertheless, both DQ and fisetin are unable to alleviate ovarian follicle depletion and subfertility caused by doxorubicin. These findings suggest that cellular senescence may not play a major role in doxorubicin-induced ovarian injury. Senolytic strategies targeting senescent cells may not be the preferred option to protect ovarian function in female cancer patients receiving doxorubicin-containing therapies, and more potent potential drugs need to be investigated.

## Data Availability

The data generated in this study are available from the corresponding authors upon reasonable request.
